# Metabolic Predictors of Cardiorespiratory Fitness Responsiveness to Continuous Endurance and High-Intensity Interval Training Programs: The TIMES Study—A Randomized Controlled Trial

**DOI:** 10.3390/metabo14090512

**Published:** 2024-09-23

**Authors:** Alex Castro, Antonio Gilberto Ferreira, Aparecida Maria Catai, Matheus Alejandro Bolina Amaral, Claudia Regina Cavaglieri, Mara Patrícia Traina Chacon-Mikahil

**Affiliations:** 1 Biosciences National Laboratory, Brazilian Center for Research in Energy and Materials, Campinas 13083-100, SP, Brazil; 2 Laboratory of Exercise Physiology, School of Physical Education, University of Campinas (UNICAMP), Campinas 13083-851, SP, Brazil; m241620@dac.unicamp.br (M.A.B.A.); cavaglie@unicamp.br (C.R.C.); 3 Laboratory of Nuclear Magnetic Resonance, Department of Chemistry, Federal University of São Carlos, São Carlos 13565-905, SP, Brazil; giba@ufscar.br; 4 Laboratory of Cardiovascular Physiotherapy, Department of Physiotherapy, Federal University of São Carlos, São Carlos 13565-905, SP, Brazil; mcatai@ufscar.br

**Keywords:** metabolomics, NMR, trainability, endurance training, high-intensity interval training

## Abstract

**Background/Objectives:** Cardiorespiratory fitness (CRF) levels significantly modulate the risk of cardiometabolic diseases, aging, and mortality. Nevertheless, there is a substantial interindividual variability in CRF responsiveness to a given standardized exercise dose despite the type of training. Predicting the responsiveness to regular exercise has the potential to contribute to personalized exercise medicine applications. This study aimed to identify predictive biomarkers for the classification of CRF responsiveness based on serum and intramuscular metabolic levels before continuous endurance training (ET) or high-intensity interval training (HIIT) programs using a randomized controlled trial. **Methods:** Forty-three serum and seventy intramuscular (vastus lateralis) metabolites were characterized and quantified via proton nuclear magnetic resonance (^1^H NMR), and CRF levels (expressed in METs) were measured in 70 sedentary young men (age: 23.7 ± 3.0 years; BMI: 24.8 ± 2.5 kg·m^−2^), at baseline and post 8 weeks of the ET, HIIT, and control (CO) periods. A multivariate binary logistic regression model was used to classify individuals at baseline as Responders or Non-responders to CRF gains after the training programs. **Results:** CRF responses ranged from 0.9 to 3.9 METs for ET, 1.1 to 4.7 METs for HIIT, and −0.9 to 0.2 METs for CO. The frequency of Responder/Non-responder individuals between ET (76.7%/23.3%) and HIIT (90.0%/10.0%) programs was similar (*p* = 0.166). The model based on serum O-acetylcarnitine levels [OR (odds ratio) = 4.72, *p* = 0.012] classified Responder/Non-responders individuals to changes in CRF regardless of the training program with 78.0% accuracy (*p* = 0.006), while the intramuscular model based on creatinine levels (OR = 4.53, *p* = 0.0137) presented 72.3% accuracy (*p* = 0.028). **Conclusions:** These results highlight the potential value of serum and intramuscular metabolites as biomarkers for the classification of CRF responsiveness previous to different aerobic training programs.

## 1. Introduction

Physical inactivity and low cardiorespiratory fitness (CRF) are associated with increased risks of non-communicable chronic diseases, especially cardiovascular disease and all-cause mortality [1,2]. CRF represents the ability of the cardiovascular and respiratory systems to provide oxygen to the muscles during exercise and can be improved primarily through aerobic training [3]. International guidelines recommend that all adults accumulate 150–300 min·week^−1^ and/or 75–150 min·week^−1^ of moderate- and vigorous-intensity cardiorespiratory exercises, respectively, to achieve a total energy expenditure ≥500 MET·min·week^−1^ [3,4].

Even following these recommendations, studies show an extraordinary interindividual variability in CRF responsiveness to standardized exercise doses, ranging from Responders and Non-responders to negative Responders [5,6,7,8]. Interestingly, there is a substantial number of individuals who do not experience a physiologically relevant increase in CRF even after traditional continuous endurance training (ET) or high-intensity interval training (HIIT) programs [5,6,9,10,11,12].

Regular exercise is indicated as an alternative to many pharmaceuticals and is recommended in the prevention and treatment of diseases. To optimize exercise prescription strategies and make physical activity part of personalized medicine, it is important to realize that, as well as pharmaceuticals, the effects of regular exercise can vary greatly between individuals [7].

Recent evidence based on omics sciences [13,14,15] supports that CRF responsiveness to traditional aerobic training regimens may be reflected by distinct molecular signatures, ranging from the gene [14,16], RNA [14,17], proteins [15,18], and metabolite levels [10,19]. Particularly, metabolites represent the dynamic signature of cellular biochemical activity, reflecting the products of interactions between the genome, transcriptome, and proteome with the cellular and tissue environment. Therefore, metabolites can provide information closer to the phenotype [20].

In this sense, metabolomics uses advanced analytical chemistry techniques to enable the high-throughput characterization of metabolites in different biological samples [21]. Particularly, nuclear magnetic resonance (NMR) has emerged as one of the main analytical techniques used in metabolomics, along with liquid chromatography or gas chromatography coupled with mass spectrometry (LC-MS and GC-MS, respectively) [22]. NMR spectroscopy stands out as a non-biased, non-destructive, readily quantifiable technique that requires easy sample preparation, enabling the detection and determination of the structure of novel compounds. NMR exhibits a high degree of automation and reproducibility, enhancing the feasibility of conducting large-scale metabolomics studies without the need for multiple internal standards for compound identification, when compared to the limitations associated with higher sensitivity and destructive methods such as LC-MS or GC-MS [22]. The GC-MS methods require chemical derivatization, are unsuitable for thermally unstable compounds with high molecular weights or for those that cannot be volatilized, and involve a complex sample preparation process [22]. In contrast, the LC-MS methods present limited reference spectral libraries, high cost of instrumentation, the need for chromatographic separation, and require extensive training [22].

NMR-based metabolomics is increasingly being used to study exercise physiology, exercise-associated metabolism [23,24,25], and, more recently, CRF responsiveness [10,19]. Studies have shown that the regulation of amino acid and carbohydrate metabolism before starting a training program [10] and during the intervention [19] is a potential mechanism for understanding the CRF responsiveness to different training programs like ET and HIIT.

Although it is not possible to predict if someone will always be a Non-responder, identifying an individual’s response potential for a specific training period can significantly guide training strategies and provide benefits to most participants, especially for Non-responder candidates. This approach helps minimize program dropout by addressing difficulties in achieving desired physiological improvements.

Studies have investigated the association between CRF responsiveness and different baseline (before training) molecular signatures. However, to date, the evidence is based on associative studies in which the sum of small effects of a large number of molecules is used to assess health risks or the explanatory contribution to variation in CRF responsiveness [16,17,18,19]. In this sense, predictive biomarkers for the classification of Responder and Non-responder individuals are largely unknown. Therefore, this study aimed to identify predictive biomarkers based on serum and intramuscular metabolic profiles for classifying CRF responsiveness prior to ET and HIIT programs.

## 2. Materials and Methods

The TIMES study (TraInability and MEtabolomicS study) is a randomized controlled clinical trial, conducted in accordance with the Declaration of Helsinki, approved by the Research Ethics Committee of the University of Campinas (CAAE: 52997216.8.0000.5404) on 12 April 2016, and included in the Brazilian Registry of Clinical Trials (ensaiosclinicos.gov.br, accessed on 21 September 2024; RBR-3rh38g). The sample, study design, and exercise training protocol of the TIMES study have been described in detail elsewhere [10,19,26]. The procedures of the experimental protocol were performed at the Laboratory of Exercise Physiology (FISEX)—School of Physical Education (FEF)—University of Campinas (UNICAMP). The acquisition and processing of the ^1^H NMR metabolomics data were conducted at the Laboratory of Nuclear Magnetic Resonance—Biosciences National Laboratory (LNBio)—Brazilian Center for Research in Energy and Materials (CNPEM).

### 2.1. Participants

Eighty healthy, young, Caucasian men were recruited through local advertisement in Campinas, Brazil, of which 70 completed the study (exercise adherence > 90%) and were considered for further analysis (age: 23.7 ± 3.0 years; height: 1.74 ± 0.06 m; body mass: 75.2 ± 8.8 kg; body fat: 20.0 ± 7.4%; body mass index: 24.8 ± 2.5 kg·m^−2^) (Supplementary Figure S1) [3,10]. Recruitment started in September 2016 and the last assessment was performed in June 2017. All participants underwent a clinical anamnesis, provided a detailed medical history, and received a medical examination that included a resting electrocardiogram. Briefly, to be included, participants need to be sedentary (not engaged in regular physical exercise defined as 30 min·week^−1^ involving an energy expenditure of ≥6 METs in the last 4 months) [3,10]. Participants were excluded if they had hypertension (blood pressure > 140/90 mmHg), diabetes (fasting glucose > 7.0 mmol·L^−1^), severe obesity (defined as body mass index > 33 kg·m^−2^), dyslipidemia (based on medication use), smoking habits, heart diseases, metabolic disorders, musculoskeletal problems interfering with exercise, or significant chronic respiratory conditions [10]. The sample size was calculated using G*Power Version 3.1.9.2 software, targeting a ~20% gain in CRF after 8 weeks of intervention, assuming a moderate effect size of Cohen’s ƒ = 0.3 for the within-between interaction design, r = 0.5 for the correlation design, and a type I error rate of 0.05 for a two-sided test, aiming for at least 80% statistical power [10].

### 2.2. Experimental Design

**Figure 1:**
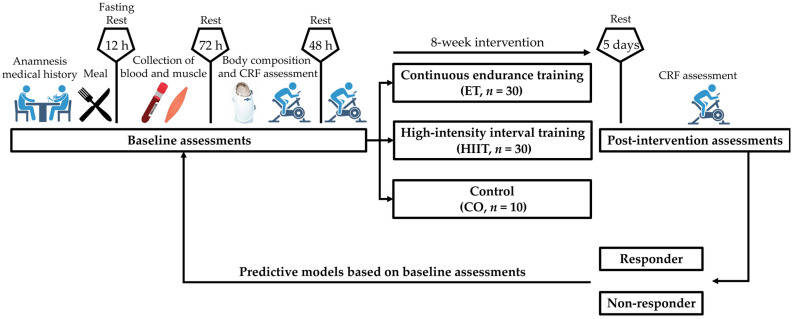
Summary of the experimental design. Participants underwent a clinical anamnesis and consumed a standardized meal. Blood and muscle tissue samples were collected 12 h later in a fasting state. After 72 h, body composition was measured using a full body plethysmograph, followed by measurements of cardiorespiratory fitness (CRF). Participants were randomly distributed into three groups: ET, HIIT, and Control (CO). CRF was reassessed after 4 weeks to adjust the training intensity and five days after the last training session to evaluate the chronic adaptations. Then participants were categorized as Responders or Non-responders to the interventions and their baseline metabolic profile was associated with CRF responses.

Prior to the intervention, the participants consumed a standardized meal, fasted for 12 h, and then blood and muscle tissue samples were collected. After 72 h, body composition was measured using a full body plethysmograph, followed by measurement of cardiorespiratory fitness, with a retest 48 h later. After collecting biological samples and analyzing body composition, 80 participants were randomly distributed into three groups in a ratio of 3:3:1 via computer-generated random numbers, of which only 70 completed the 8 weeks of intervention and were considered for further analysis: ET (*n* = 30), HIIT (*n* = 30) and Control (CO, *n* = 10) (Figure 1). This unequal randomization strategy was used since the analysis of Responders and Non-responders to training programs was based only on the intervention groups, avoiding additional and unnecessary invasive assessments in the control group. CRF was reassessed after 4 weeks to adjust the training intensity and five days after the last training session to evaluate the chronic adaptations [10].

### 2.3. Standardization of Meals Prior to Data Collection

Before collecting blood and muscle tissue (12 h prior), participants consumed a standardized meal with 55–60% carbohydrate, 20–25% lipids, and 15–20% protein, with an energy value corresponding to 30% of the total individual energy expenditure estimated in order to avoid the effects of dietary variations on the blood and muscle metabolic profile [27,28].

### 2.4. Blood and Muscle Tissue Samples

Venous blood and muscle tissue samples were collected between 7 and 10 am, fasting for 12 h. Participants were instructed to refrain from caffeine consumption for a minimum of 12 h, as well as from alcohol consumption and exercise for the preceding 48 h. Blood samples were kept at rest for 30 min, centrifuged at 5000 rpm for 10 min, and then serum aliquots were collected and stored in a freezer at −80 °C. After blood collection, muscle tissue was obtained via lateral vastus biopsy from the quadriceps of the individual’s dominant limb. The collection was carried out after trichotomy and asepsis of the region. A small portion of the area was anesthetized with 2% xylocaine through a subcutaneous injection, and an incision (~5 mm) was made to the muscular fascia using a scalpel. A Bergström needle was then inserted into the muscle (~3 cm) to obtain the sample. After the tissues’ removal, the incision was closed and covered with sterile bandages. All extracted muscle samples were cleaned (freed of blood and excess connective tissue), aliquoted, immediately frozen in liquid nitrogen, and stored at −80 °C for further analysis [10].

### 2.5. Body Composition and Cardiorespiratory Assessments

Body composition was assessed via a calibrated whole-body plethysmograph (BOD POD^®^, Body Composition System, Life Measurement Instruments, Concord, CA, USA) with participants wearing no shoes or outer garments. All participants were asked not to eat or exercise for 2 h prior to assessment [10].

CRF was evaluated during a progressive incremental test until exhaustion using a calibrated electromagnetic brake cycle ergometer (Corival 400, Quinton^®^ Instrument Co., Groningen, The Netherlands), with a retest after 48 h. After 5 min at rest, the test started with 3 min of warm up at 50 W, followed by increments of 25 W·min^−1^ until voluntary exhaustion. The pedaling rate was 70−80 rpm. The test was interrupted when the participant was unable to continue or maintain a minimum rate of 70 rpm despite verbal encouragement [10]. Heart rate (HR) was recorded at rest and continuously throughout the test using the heart rate monitor (S810, Polar^®^, Kempele, Finland), while the subjective perception of effort was recorded at the end of each stage of the progressive test using the 6–20 Borg scale [29].

The participants’ maximal power output (MPO) was estimated as W + [25·(*t*/60)], where W is the last load reached and *t* is the number of seconds in the test’s final load. The highest MPO value recorded between tests was defined as the measure of CRF [10,19,26]. For greater applicability of the results, CRF was expressed in metabolic equivalent units (METs) as previously described [3]. The coefficient of variation (CV) and intra-class correlation coefficient (ICC) for the CRF test-retest measurements were 2.8% and 0.98, respectively.

### 2.6. Exercise Training Programs

Throughout the training program, the exercise intensity was individualized and customized for each participant based on heart rate reserve (HRr) [30]. Both ET and HIIT programs were designed to obtain the same exercise volume in total and by session. A complete and detailed description of training volume balancing between groups can be found elsewhere [10]. Briefly, the intervention was composed of the following: ET [40 min at 70–75% HR reserve (HRr), 3–4 d·week^−1^] and HIIT [40 min (5 sets of 4 min at 90% HRr interspersed with sets of 3 min at 50–60% HRr), 3–4 d·week^−1^] exercise programs, while the CO group did not perform exercises, over a period of 8 weeks. After 4 weeks, individuals from the CO group were contacted and reminded of the importance of maintaining a sedentary lifestyle and informed about the schedule of the post-program testing visits.

All exercise sessions were supervised to ensure that the cycling cadence (70–80 rpm) and target HR were maintained. In all training sessions, the workload of the cycle ergometer was adjusted manually to consider the variation in HR response for each participant. All participants were encouraged to drink water ad libitum, and the environmental temperature was kept around 21–23 °C. The training intensity was adjusted in the 5th week. In the end, 48 h after the last training session, all assessments performed in the pre-training were repeated.

### 2.7. Sample Preparation for Metabolomics Analysis

Blood serum and muscle tissue samples were prepared for metabolomic analysis as described in previous studies [10,19,26]. Briefly, 500 μL of blood serum was added to pre-washed 3 kDa filters (Amicon Ultra, Millipore^®^, Darmstadt, Germany) and centrifuged at 14,000 rpm for 45 min at 4 °C for macromolecule removal. Then, 250 μL of filtered serum was transferred to a 5 mm NMR tube (Standard Series 5 mm, Wilmad^®^, Vineland, NJ, USA) containing 60 μL of phosphate buffer 0.1 M [(monobasic sodium phosphate, NaH_2_PO_4_ H_2_O, 137.99 g·mol^−1^; dibasic sodium phosphate, Na_2_HPO_3_, 141.96 g·mol^−1^), TMSP-d_4_ (3-(trimethylsilyl)-2,2′,3,3′-tetradeuteropropionic acid)] and 290 μL of D_2_O (99.9%; Cambridge Isotope Laboratories Inc.^®^, Andover, MA, USA). The concentration of the internal reference (TMSP-d_4_) in the NMR tube was 0.5 mmol·L^−1^. For muscle tissue, ~40 mg fragments were weighed and added to a cold methanol/chloroform solution (2:1 *v*/*v*, total 2.5 mL). Tissues were homogenized on ice (3 cycles of 30 s, alternated with a 10 s pause) and sonicated (3 cycles of 1 min, alternated with a 10 s pause). After that, a cold solution of chloroform/Milli-Q water (1:1 *v*/*v*, total 2.5 mL) was added to the samples. The samples were briefly vortexed to form an emulsion and centrifuged at 2000× *g* for 30 min at 4 °C. The upper phase of the mixture containing methanol, water, and polar metabolites was collected and evaporated in a vacuum concentrator (miVac Duo Concentrator, Genevac^®^, Ipswich, UK). The remaining solid phase was rehydrated in 0.6 mL of deuterium oxide containing a phosphate buffer (0.1 M, pH 7.4) and 0.5 mM TMSP-d_4_ and then it was added to a 5 mm NMR tube (Standard Series 5 mm, Wilmad^®^, Vineland, NJ, USA), for immediate acquisition on the NMR spectrometer.

### 2.8. NMR-Based Metabolomics, Data Acquisition, and Metabolite Characterization

^1^H NMR spectra were acquired using Vnmr Version 4.2 software on an Inova Agilent spectrometer 600 MHz (Agilent Technologies Inc., Santa Clara, CA, USA) equipped with a 5 mm TCI cryoprobe and operating at a constant temperature of 298 K (25 °C). A total of 256 free induction decays (FID) with 32-k data points over a spectral width of 8000 Hz were used, with an acquisition time of 4 s and 1.5-s intervals between scans (relaxation delay), and a 90° pulse of 6.2 μs with a water suppression pulse sequence. All acquired spectra were processed using Chenomx NMR Suite^®^ Version 8.6 software (Chenomx Inc., Edmonton, AB, Canada), applying phase adjustment, baseline correction using the auto spline algorithm and manual adjustment, removal of water signal (4.6–5.1 ppm), shimming correction, line broadening (lb) of 0.5 Hz, and spectral calibration. The characterization and quantification of the metabolites were based on the Chenomx library and the signal of TMSP-d_4_ (internal reference) at ~0.00 ppm, respectively. Also, 2D Hetero Single Quantum Coherence (HSQC) and TOtal Correlation SpectroscopY (TOCSY) experiments were used to assist in the characterization of the most relevant compounds (Supplementary Figures S2 and S3). The followed parameters were assumed for the HSQC experiment: SWF1 200 ppm and SWF2 13 ppm, relaxation delay = 1.5 s, number of experiments = 112 for serum and 128 for skeletal muscle, TDF1 = 256 points and TDF2 = 2048, with an acquisition time of 0.128 s; and for the TOCSY experiment: SWF1 and SWF2 = 13 ppm, relaxation delay = 1.5 s, number of experiments = 40 for serum and 64 for skeletal muscle, TDF1 = 512 points and TDF2 = 2048 points, with an acquisition time of 0.128 s.

The intramuscular methanol and serum ethanol metabolites were not considered for further analysis as they were biased due to reagents used in the collection and preparation of samples. Additionally, serum 2-Aminobutyrate, 2-Hydroxybutyrate, 2-Oxoglutarate, Acetate, Acetoacetate, Fumarate, Glucose, Methylamine, and Oxypurinol metabolites were not considered for further analysis, as they were judged to have low reproducibility (coefficient of variation < 25% and intraclass correlation coefficient < 0.75), as previously reported [10]. The median coefficient of variation and intraclass correlation coefficient of serum metabolites were 8.5% (3–23%) and 0.98 (0.79–1.00), respectively.

### 2.9. Statistical Analysis

To verify whether the variability in CRF responses promoted by training programs was significantly higher than those expected to occur in the absence of training (random error) [6,31,32], individual responses standard deviation (SDIR) with a 95% confidence interval (95% CI) were calculated according the following formulas: SDIR = $\surd {SD}_{EXP}^{2}$ − $\surd {SD}_{CON}^{2}$ and SDIR ± 1.96×$\surd [2\times ({SD}_{EXP}^{4}/{SD}_{EXP})$ + $\left({SD}_{CO}^{4}n/{SD}_{CO})\right]$ for 95% CI estimates, where SD_EXP_ is the observed interindividual variability in CRF changes in the exercise group (ET or HIIT) and SD_CON_ is the observed interindividual variability in CRF changes in the control group [33]. Thus, if the confidence intervals for the SDIR did not cross zero, the interindividual variability of CRF responses is indeed induced by the training programs. The frequency of occurrence of Responders and Non-responders between the training programs was explored via Chi-square test.

To explore the main metabolites associated with CRF responsiveness and to reduce data dimensionality, simple binary logistic regression was conducted. Afterward, metabolites significantly associated with CRF responsiveness were retained for a multivariate binary logistic regression analysis with the forward (Wald) method for variable selection, assuming the level of responsiveness (Responder = 1 or Non-responder = 0) as the dependent variable. Age, body mass index, body fat, baseline CRF, type of training (HIIT = 1, ET = 0), and pre-training serum and intramuscular metabolic levels were the predictive variables. Metabolite levels were standardized to mean = 0 and multiples of 1 standard deviation (Z-score) [18]. CRF responsiveness was based on minimal clinically important difference (MCID = 1 MET) and technical error (TE = 0.3 METs) estimated with 95% CI [6]. Briefly, TE was calculated by computing the within-participant standard deviation from repeated measures of the baseline CRF assessments (Figure 1), divided by $\sqrt{2}$ [34]. We used 1 MET as the MCID based on evidence that it is associated with a 15–20% decreased risk of all-cause and cardiovascular disease mortality [35,36]. Combining the MCID and TE improves the confidence in the Responders/Non-responders classifications [11]. Participants were classified as Responders for changes (post–pre-training) in CRF higher than MCID + (1.96×TE) or as Non-responders for changes lower than or equal to MCID − (1.96×TE). Thus, if the 95% confidence interval for individual responses includes values less than 1 MET, the individual is considered a Non-responder. To be classified as a Responder, the 95% confidence interval must include only values above 1 MET. The general metabolomic signature between Responders and Non-responders was presented using an ilustrative heatmap. The accuracy of the predictive model was analyzed by the receiver operating characteristic curve (ROC curve), the area under this curve, sensitivity (true positive rate of Non-responders), specificity (true positive rate of Responders), and odds ratio (OR). The discriminant cut-off values between Responders and Non-responders were obtained via Youden index analysis [37]. In addition, to compare the baseline metabolic levels between Responders and Non-responders for the compounds retained in the regression models, an analysis of variance adjusted by type of training was conducted. All analyzes were performed using the SPSS^®^ statistics Version 25.0 software (SPSS Inc.^®^, Chicago, IL, USA). The significance level was set at 5%.

## 3. Results

### 3.1. Participant Characteristics and Variability of Responses

There were no significant differences in participants’ characteristics among ET, HIIT, and CO groups (Table 1). There was a significant increase in CRF for both ET (average change = +2.1 MET, *p* < 0.001) and HIIT (average change = +2.6 MET, *p* < 0.001) groups, with no changes for the CO (average change = −0.4 MET, *p* = 0.077) group. In addition, CRF levels at post-training were higher in ET and HIIT groups compared to the CO group (*p* < 0.001 for both).

**Figure 2:**
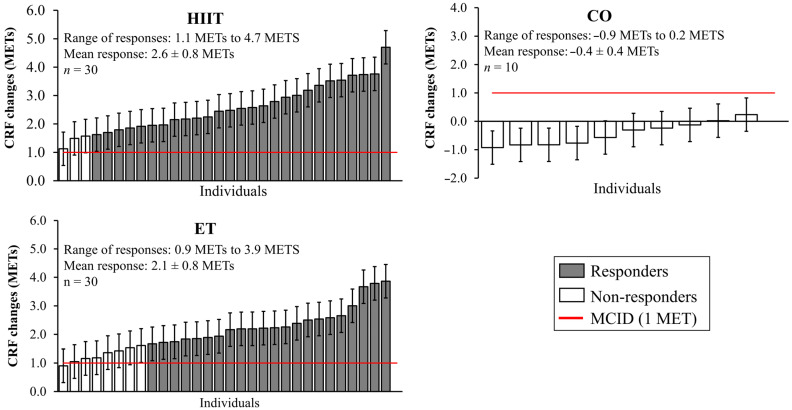
Distribution of the CRF individual responses ± 95% confidence intervals (95% CIs) in relation to the minimal clinically important difference (MCID) for continuous endurance training (ET), high-intensity interval training (HIIT), and the control group (CO) in TIMES. The 95% CIs are calculated as the observed response ± 1.96 × (technical error). The dashed line represents the MCID [1 multiple of the resting metabolic rate (MET)].

The variability in CRF responses promoted by ET and HIIT were significantly higher than those expected to occur in the absence of training: SDIR (ET vs. CO) = 0.64 METs (95% CI: 0.31 to 0.98 METs); SDIR (HIIT vs. CO) = 0.74 METs (95% CI: 0.34 to 1.14 METs). However, there was no difference between training programs: SDIR (HIIT vs. ET) = 0.36 METs (95% CI: −0.11 to 0.84). Additionally, there were no significant differences in the frequency of Responders and Non-responders (*p* = 0.166, χ^2^ = 1.920) between ET [Responders: 23 (76.7%); Non-responders: 7 (23.3%)] and HIIT [Responders: 27 (90.0%); Non-responders: 3 (10.0%)] programs (Figure 2).

### 3.2. Predictive Models

**Figure 3:**
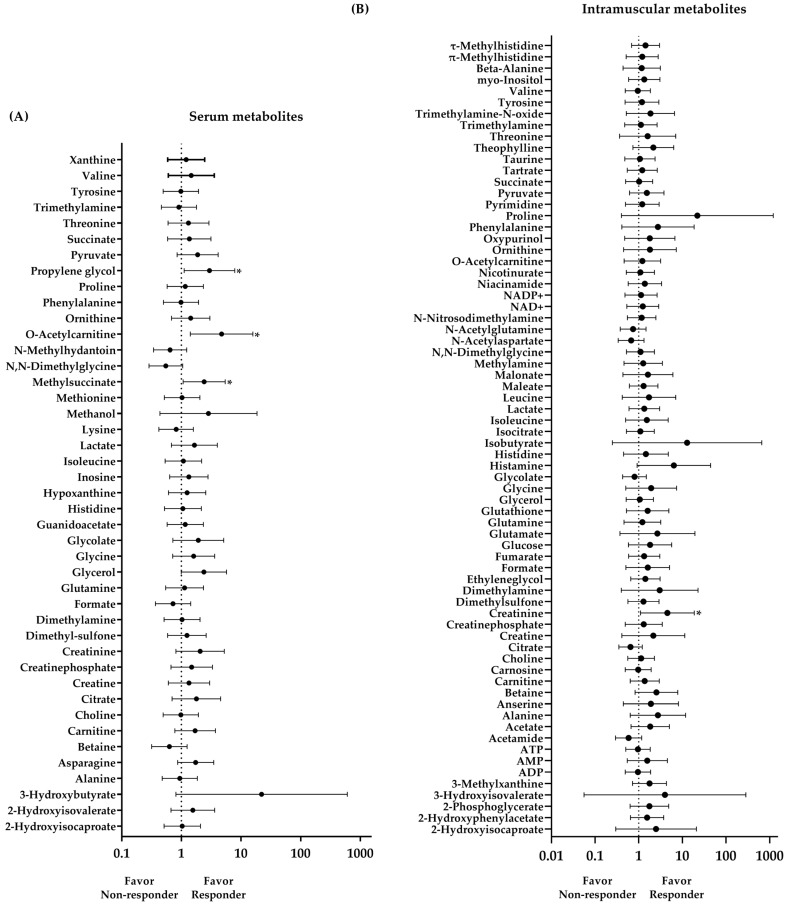
Odds ratio for the classification of CRF responsiveness based on baseline serum (**A**) and intramuscular (**B**) metabolite levels. The x-axis is represented on a logarithmic scale. * *p* < 0.05.

A total of 43 and 70 metabolites were characterized and quantified in serum and muscle, respectively. The CRF responsiveness was associated with the serum O-Acetylcarnitine (OR = 4.72; *p* = 0.012), Methylsuccinate (OR = 2.41; *p* = 0.034), and Propylene glycol (OR = 2.95; *p* = 0.030) levels, as well as intramuscular creatinine (OR = 4.53; *p* = 0.037) levels (Figure 3). Higher levels of these metabolites indicate a greater chance of being a Responder to any training program.

**Figure 4:**
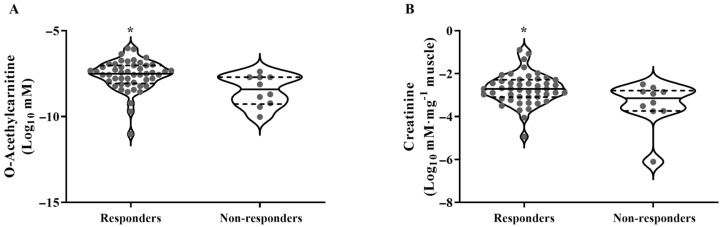
Violin plots of baseline metabolite levels for the compounds significantly retained in the models. Data are individual values (log-transformed data) and horizontal lines representing the median (solid center line) and first and third quartiles (dashed lines); * *p* < 0.05 when compared to Non-responders, adjusted for the training programs. At the baseline, Responders showed higher levels of (**A**) serum O-acetylcarnitine and (**B**) intramuscular Creatinine compared to Non-responders (*p* = 0.012 and *p* = 0.017).

**Figure 5:**
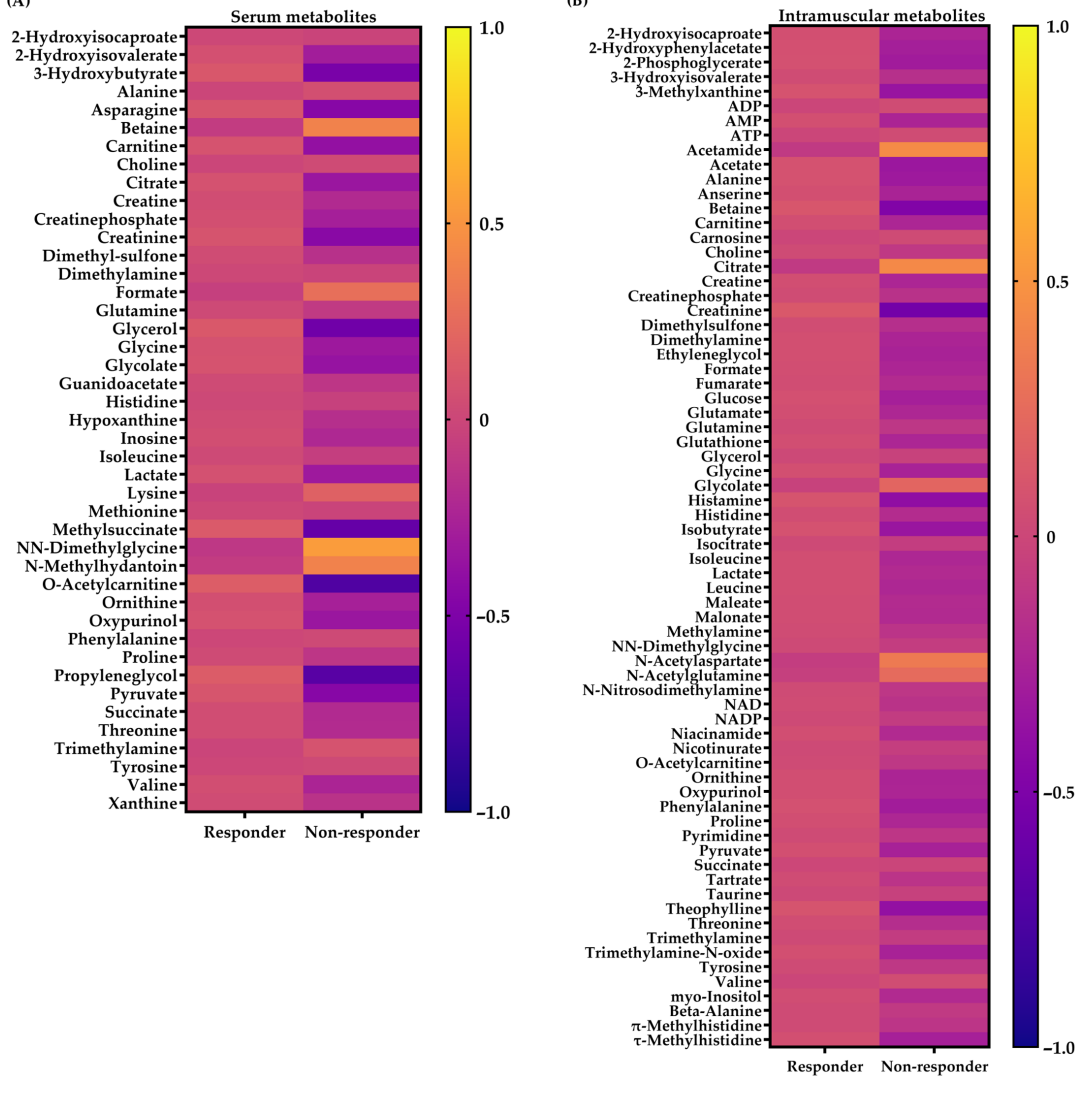
Baseline metabolomic signature between Responders and Non-responders regardless of the training group [*n* = 59 for serum (**A**) and *n* = 57 for muscle (**B**)]. Cells represent the mean standardized concentration levels (z-score) for each metabolite (lines) between groups (columns). Yellow and blue colors represent higher and lower concentration levels, respectively.

From these metabolites, two models were created (Table 2). Model 1 is as follows: Serum O-acetylcarnitine levels classified Responders and Non-responders with 78.0% accuracy (*p* = 0.006; sensitivity = 90.0%; specificity = 61.2%). Each one-unit increase in serum O-acetylcarnitine levels increases the chance of being a Responder by 4.72 times. A cutoff value of 0.87 was used, with lower values indicating a higher chance of being a Non-responder and higher values indicating a higher chance of being a Responder. Model 2 is as follows: Intramuscular creatinine levels classified Responders and Non-responders with 72.3% accuracy (*p* = 0.028; sensitivity = 80.0%; specificity = 57.4%). Each one-unit increase in intramuscular creatinine levels increases the chance of being a Responder by 4.53 times. A cutoff value of 0.83 was used, with lower values indicating a higher chance of being a Non-responder and higher values indicating a higher chance of being a Responder. Age, body mass index, body fat, baseline CRF, and type of training did not significantly affect CRF responsiveness for any of the models (*p* > 0.05 for all). Therefore, the metabolomic signature between Responders and Non-responders, as well as subsequent analyses, was presented regardless of the training group. In addition, at the baseline, Responders showed higher levels of serum O-acetylcarnitine and intramuscular Creatinine compared to Non-responders (*p* = 0.012 and *p* = 0.017, Figure 4). The complete metabolic signature between Responders and Non-reponders is presented in Figure 5.

## 4. Discussion

This study aimed to identify predictive biomarkers based on serum and intramuscular metabolic levels for the classification of CRF responsiveness prior to ET and HIIT programs. The main findings were as follows: (i) there was no significant difference in the frequency of Responders and Non-responders between ET and HIIT programs; (ii) the metabolic model based on pre-training serum O-acetylcarnitine levels, as well as intramuscular creatinine levels, was able to classify Responder and Non-responder individuals for CRF gains, regardless of the training program, with 78.0% and 72.3% accuracy, respectively; and (iii) interestingly, the less invasive model (serum metabolites) showed slightly higher accuracy for the classification of CRF responsiveness.

We observed no difference in the proportion of Responders and Non-responders between ET and HIIT programs. These results can be explained by both training programs being perfectly matched for time, work per session, and total volume of exercise. Our results suggest that for sedentary people, ET has the potential to induce as much improvement in CRF as HIIT programs [10,11], as well as promote similar variability in CRF responses [SDIR for HIIT vs. ET = 0.36 METs (95% CI: −0.11 to 0.84)]. Furthermore, both the ET and HIIT groups exhibited greater variability in CRF responses compared to the CO, suggesting that the observed variation in responses within the training programs is higher than that which would be expected to occur due to random factors [6].

In the serum model, higher O-acetylcarnitine levels were linked to an increased likelihood of being classified as a Responder, regardless of the training program. O-acetylcarnitine is crucial for transporting long-chain fatty acids into the mitochondria for oxidation and energy production. While the exact role of serum/plasma O-acetylcarnitine is unclear, it may reflect the traffic between cells and organs, cellular and tissue acylcarnitine sequestration [13,38], or even increased fatty acid oxidation in muscle [38,39,40]. Supporting our findings, previous studies have shown that baseline serum/plasma levels of O-acetylcarnitine and several medium- and long-chain fatty acyl-carnitines (C6-, C7-, and C16-acylcarnitines) are positively correlated with improvements in submaximal [13] and maximal [10,41] CRF in response to different aerobic training programs. Thus, higher baseline levels of circulating O-acetylcarnitine may indicate greater acetylation capacity in Responder individuals [13,41].

In the context of the intramuscular model, higher creatinine level was also associated with an increased chance of classification as a Responder, regardless of the training program. Creatinine is a breakdown product of creatine and phosphocreatine that is a source of energy for skeletal muscle contraction [42]. Although intramuscular creatinine levels have been associated with higher CRF gains [10], its functional relation with the regulation of CRF responsiveness is still not well understood. Creatinine diffuses out of the cells into the blood and is excreted by the kidneys into the urine [43]. Surprisingly, serum creatinine did not show a significant association with changes in CRF and therefore was not considered suitable for inclusion in the serum metabolite model. It is likely that Responders might benefit from more efficient creatine metabolism, which enhances creatinine excretion while maintaining balanced serum levels. This is supported by studies showing a positive association between urine creatinine and baseline CRF levels [44,45], since that creatinine excretion in urine is directly proportional to the creatine content in muscle [46]. According to this, in our study the intramuscular creatinine levels were positively correlated to creatine levels (r = 0.678, *p* < 0.001).

In both serum and intramuscular models, higher metabolic values were associated with a greater likelihood of being a Responder. However, one metabolite, serum N,N-Dimethylglycine, showed a trend (OR = 0.548, *p* = 0.070) indicating that higher values were associated with an increased chance of being a Non-responder. N,N-Dimethylglycine levels have been negatively correlated with CRF gains in response to different aerobic training programs through the regulation of glycine, serine, and threonine metabolism [10]. N,N-Dimethylglycine is associated with reduced oxidative stress [47]. Since exercise-induced oxidative stress is positively linked to improvements in endurance adaptations [48], individuals with higher levels of N,N-Dimethylglycine may be less likely to improve CRF.

Curiously, only few metabolites were retained in the models. On the other hand, a simpler model is likely more practical. While previous studies have shown models composed of many molecules contributing small effects to the associations between baseline molecular profiles and CRF gains [16,18,49], this is the first study to propose a metabolism-based classification model. Moreover, this study robustly determined Responders and Non-responders by considering both clinically important changes and the estimation of the 95% range of variability for each individual’s response. We believe that increasing the sample size, especially for Non-responders, and incorporating additional analytical platforms such as GC-MS and LC-MS could help identify more CRF-related metabolites, allowing the construction of a more elucidative model. 

The presented models increased the chances of correctly identifying a Non-responder individual from 18.3% (null model) to 80–90% (serum and intramuscular models), as observed for the sensitivity values in ROC curve analysis. These individuals would benefit most from personalized training, making their prior identification particularly valuable since they would be more susceptible to cardiovascular disease and all-cause mortality. In terms of implementation, muscle biopsies offer specific biomarkers for CRF adaptations, but are invasive and complex. In contrast, the serum model, though reflecting more general adaptations from multiple systems, provides comparable accuracy and is easier to implement than the muscle model. However, both models have low specificity and should be interpreted with caution.

These findings highlight new predictor biomarkers of CRF responsiveness. Identifying these biomarkers with NMR can help classify individuals as Responders or Non-responders before starting a training program. This advance has the potential for the development of biologically personalized exercise prescriptions and portable devices for measuring specific compounds, similar to how glucose, lactate, and others are currently measured by physiologists in gyms, clinics, and the field during training routines.

We recognize some limitations in the present study that should be considered further. The studied sample was composed only of young men; therefore, extrapolation of the results to other populations should be avoided. Unfortunately, women were not selected for participation in the study due to the significant influence of menstrual cycle variations on our primary metabolic measurements [50]. Given our limited resources and the need to concentrate participants on a single assessment day, it was not feasible to accommodate the different collection times required for these variations. Attaining complete control over dietary intake proves to be a challenging endeavor for free-living individuals engaged in exercise training studies. The training programs were of relatively short duration and may not have been sufficient to promote the maximal training response in all individuals, given the exercise dose considered in this study [51,52]. Only two training programs were tested, which may not reflect the variability of responses expected for other training protocols, such as sprint interval training programs [53]. Studies suggest that very long periods of training, with higher intensity and weekly volume, may maximize individual responses [5,52], which may also decrease adherence to the program, especially for sedentary individuals. The exercise prescription was not based on physiological thresholds but rather on a maximal physiological index [28,54]. The number of Non-responders was low, especially for the HIIT group. However, the type of training did not significantly improve the model’s fit, as the frequency of Responders vs. Non-responders was not statistically different between groups. This suggests that the metabolites retained in the models are linked to CRF responsiveness regardless of the training type. This outcome is likely due to both training programs being matched in total exercise volume and average intensity, leading to similar CRF gains between groups after training [10].

On the other hand, our study also presents some strengths, including the substantial sample size that involved serum and intramuscular data. The diet control employed—including the consumption of a standardized meal and 12 h of fasting before the collection of blood and muscle tissue samples—is expected to minimize metabolic differences between participants [28]. The use of the NMR-based metabolomics approach allowed for exploring new biomarkers, providing quantitative thresholds for different metabolites to classify Responder and Non-responder individuals prior to training programs. Finally, the criterion adopted to define Responder and Non-responder accounted for MCID and other sources of error, providing greater practical validity for our findings.

## 5. Conclusions

The findings of this study highlight the potential value of serum and intramuscular metabolites as biomarkers for classifying CRF responsiveness to different aerobic training programs. Our results show that predictive models using serum O-acetylcarnitine and intramuscular creatinine can classify individuals as Responders or Non-responders to CRF gains with reasonable accuracy, regardless of the training program (ET or HIIT), with the serum model showing slightly higher accuracy. These metabolites may provide insights into metabolic pathways important for regulating CRF responsiveness. Future studies are encouraged to investigate the sources of these metabolites, their functional significance for CRF, and to replicate our metabolic models in other participant cohorts.

## Figures and Tables

**Table 1:** Baseline characteristics of participants and changes in CRF and heart rate Pre and Post the interventions.

Variables		ET (*n* = 30)			HIIT (*n* = 30)			CO (*n* = 10)		
*Characteristics of the participants*										
Age (years)	Pre	23.3	±	3.4	23.5	±	2.6	23.6	±	3.5
Height (m)	Pre	1.7	±	0.1	1.7	±	0.1	1.7	±	0
Body mass (kg)	Pre	72.1	±	12.1	72.1	±	10.3	76.5	±	8.4
Body fat percentage (%)	Pre	20.3	±	7.3	21.3	±	7.6	21.6	±	5.7
BMI (kg m^2^)	Pre	23.9	±	3.4	23.8	±	2.7	25	±	2.6
MPO (W)	Pre	237.5	±	38.4	237.6	±	32.4	249.5	±	28.7
*Changes Pre to Post intervention*										
HR_MAX_ (beats min^−1^)	Pre	192	±	9	192	±	8	195	±	9
	Post	192	±	7	191	±	7	195	±	12
CRF (MET) ^a^	Pre	12.3	±	1.9	12.3	±	1.8	12.1	±	1.1
	Post	14.4	±	1.9 *^†^	14.9	±	2.0 *^†^	11.7	±	1.0

ET: Continuous endurance training; HIIT: High-intensity interval training; CO: Control; Body mass index; MPO: Maximal power output; HR_MAX_: Maximal heart rate; CRF: Cardiorespiratory fitness; MET: Metabolic equivalent; ^a^ Significant interaction group*time (*p* < 0.001). * Difference from Pre (*p* < 0.01). ^†^ Difference from CO group (*p* < 0.01). Adapted from Castro et al. [10].

**Table 2:** Multivariate binary logistic regression models for the classification of CRF responsiveness.

Models	Predictive Variables	*B*	*p*-Value	OR (95% CI)			
Model 1 (*n* = 59)	O-acetylcarnitine (serum)	1.55	0.012	4.72	(1.40	to	15.80)
	Constant	2.15	<0.001	8.60	--------------		
Model 2 (*n* = 57)	Creatinine (intramuscular)	1.51	0.037	4.53	(1.09	to	18.70)
	Constant	1.99	<0.001	7.35	--------------		

OR: Odds ratio indicating the chance of occurrence of responder individuals from the predictive variables; 95% CI: 95% confidence interval. *B*: Regression coefficient; Mean ± standard deviation for each metabolite, before z-score standardization: serum O-acetylcarnitine = 0.0055 ± 0.0031 mM and intramuscular creatinine = 0.1618 ± 0.0935 mM·g^−1^.

## Data Availability

The original contributions presented in the study are included in the article/Supplementary Materials. Further inquiries can be directed to the corresponding authors.

## Supplementary Materials

The following supporting information can be downloaded at: https://www.mdpi.com/article/10.3390/metabo14090512/s1, Figure S1. Flow diagram of participants in the TIMES study [10]. Figure S2. ^1^H NMR spectrum with the most relevant serum (A) and intramuscular (B) metabolites annotated, acquired at 14.1 T, 25 °C in D2O. TMSP-d4 was used as internal reference.; Figure S3. 2D Hetero Single Quantum Coherence (HSQC) experiment for skeletal muscle (A) and serum (C) samples and TOtal Correlation SpectroscopY (TOCSY) experiment for skeletal muscle sample (B) with the most relevant metabolites annotated. No correlation was noted for the serum creatinine and O-acetylcarnitine in the TOCSY experiment.
