# Supplementary material for: Metabolic Predictors of Cardiorespiratory Fitness Responsiveness to Continuous Endurance and High-Intensity Interval Training Programs: The TIMES Study—A Randomized Controlled Trial
Source: Metabolites. 2024 Sep 23;14(9):512. doi: 10.3390/metabo14090512 (PMC11433752; doi:10.3390/metabo14090512)
Supplement: Supplementary file 1 [file metabolites-14-00512-s001.zip › metabolites-3167163-supplementary.pdf]

metabolites

MDPI

Metabolic Predictors of Cardiorespiratory Fitness Responsiveness to Continuous Endurance and High-Intensity Interval Training Programs: The TIMES Study - A Randomized Controlled Trial

Alex Castro [1,2,3]*, Antonio Gilberto Ferreira [3], Aparecida M. Catai [4], Matheus Alejandro Bolina Amaral [2], Claudia Regina Cavaglieri [2] and Mara Patrícia Traina Chacon-Mikahil [2].

1 Biosciences National Laboratory, Brazilian Center for Research in Energy and Materials, Campinas, SP, 13083-100, Brazil; ax.castro@yahoo.com.br and alex.castro@lnbio.cnpem.br (A.C.)
2 Laboratory of Exercise Physiology, School of Physical Education, University of Campinas (UNICAMP), Campinas, SP, 13083-851, Brazil; m241620@dac.unicamp.br (M.A.B.A.); cavaglie@unicamp.br (C.R.C.)
3 Laboratory of Nuclear Magnetic Resonance, Department of Chemistry, Federal University of São Carlos, São Carlos, SP, 13565-905, Brazil; giba@ufscar.br
4 Laboratory of Cardiovascular Physiotherapy, Department of Physiotherapy, Federal University of São Carlos, São Carlos, SP, 13565-905, Brazil; mcatai@ufscar.br
* Correspondence: ax.castro@yahoo.com.br and alex.castro@lnbio.cnpem.br (A.C.); marapatricia@fef.unicamp.br (M.P.T.C.-M.)

Supplementary Material

metabolites

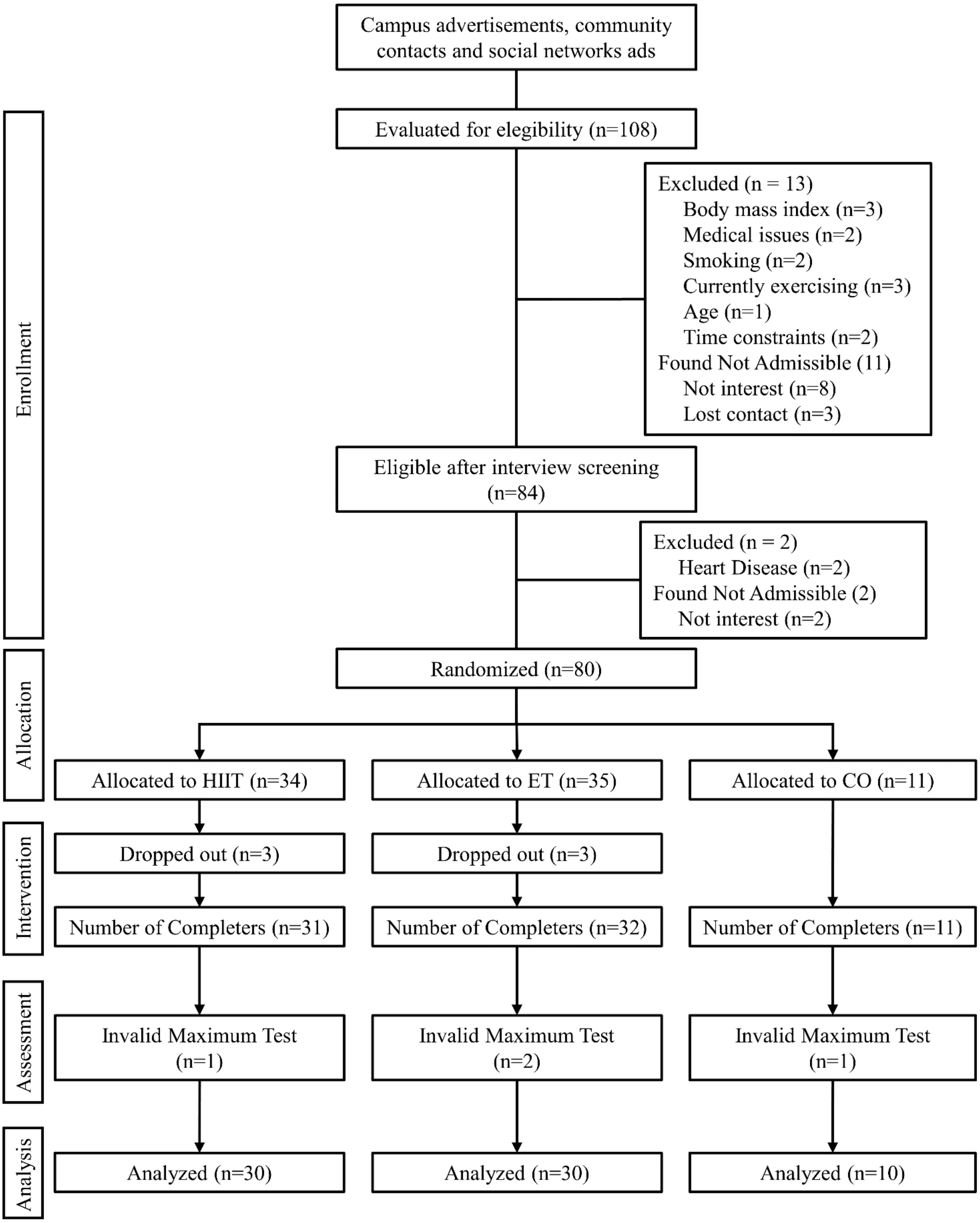

Figure S1. Flow diagram of participants in the TIMES study [10].

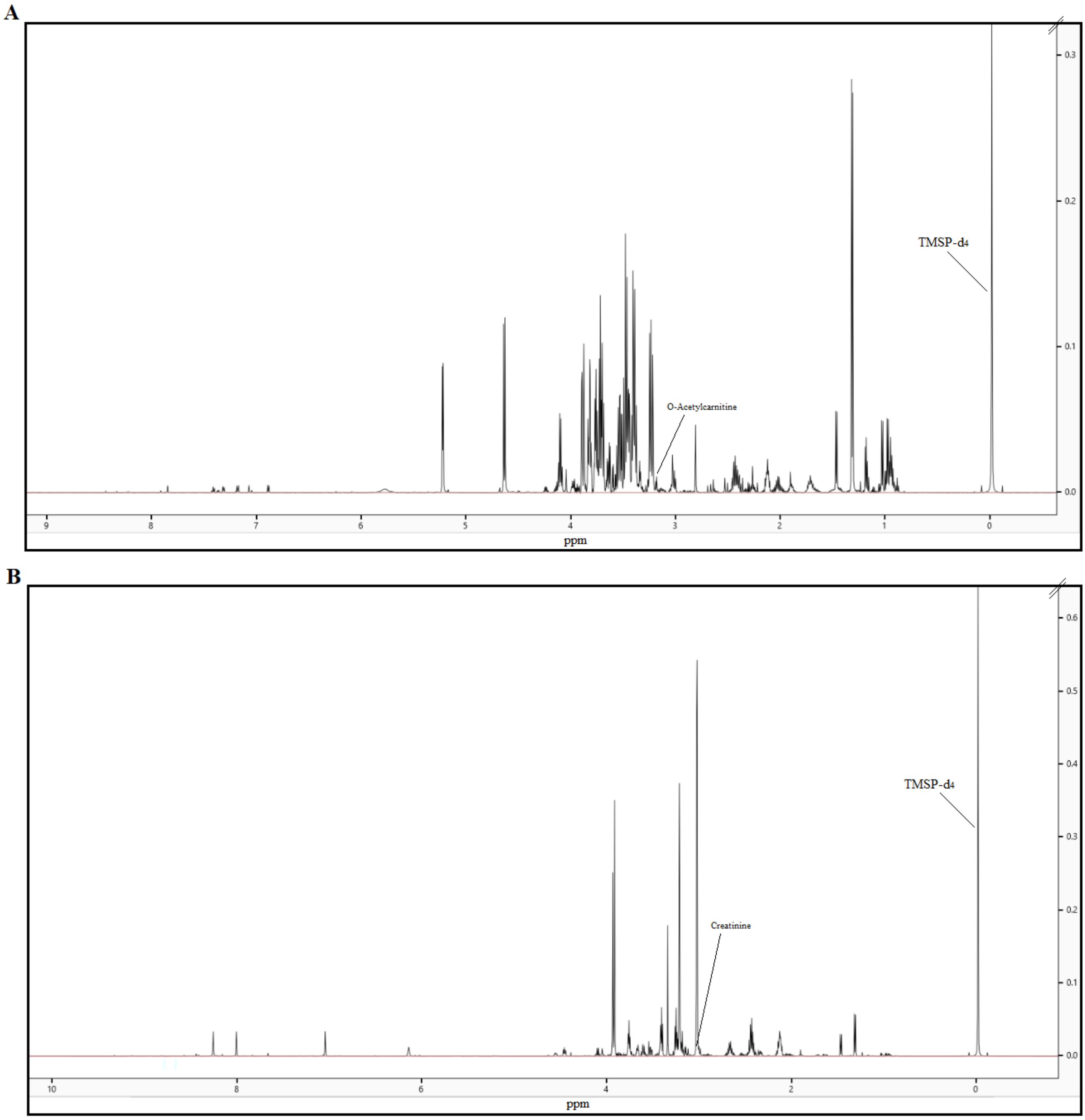

Figure S2. ^{1}H NMR spectrum with the most relevant serum (**A**) and intramuscular (**B**) metabolites annotated, acquired at 14.1 T, 25 ºC in D_2O. TMSP-d4 was used as internal reference.

metabolites

MDPI

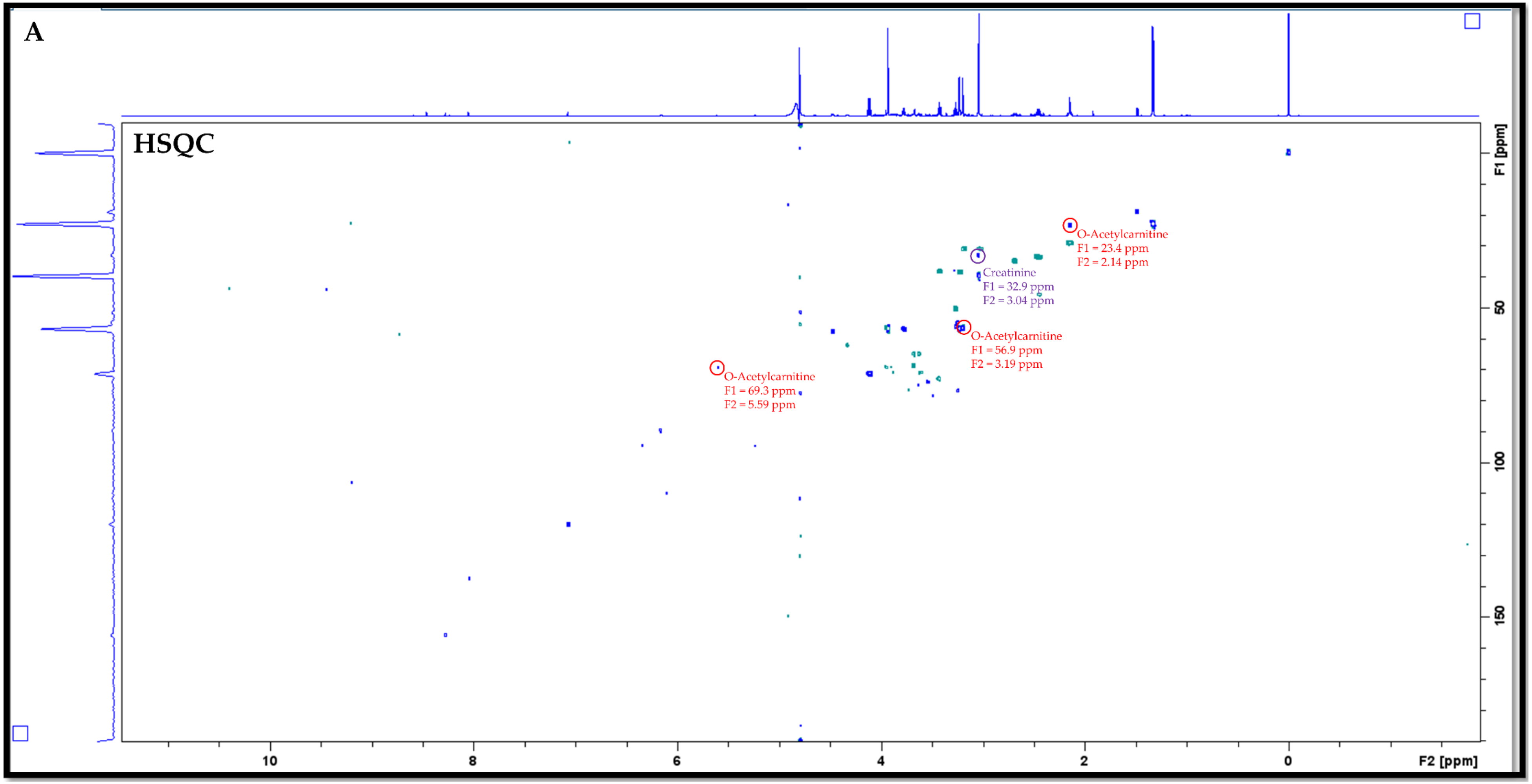

metabolites
MDPI

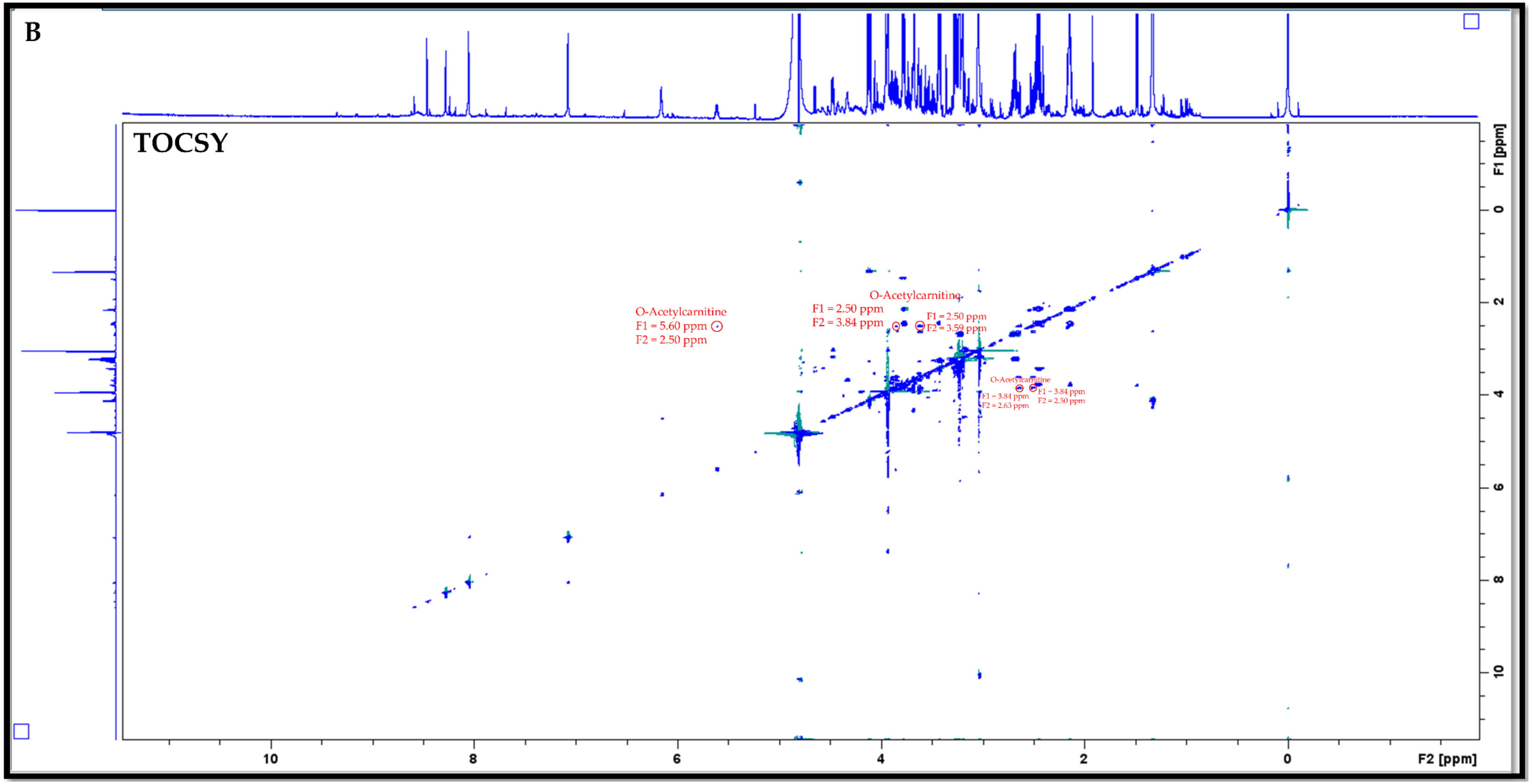

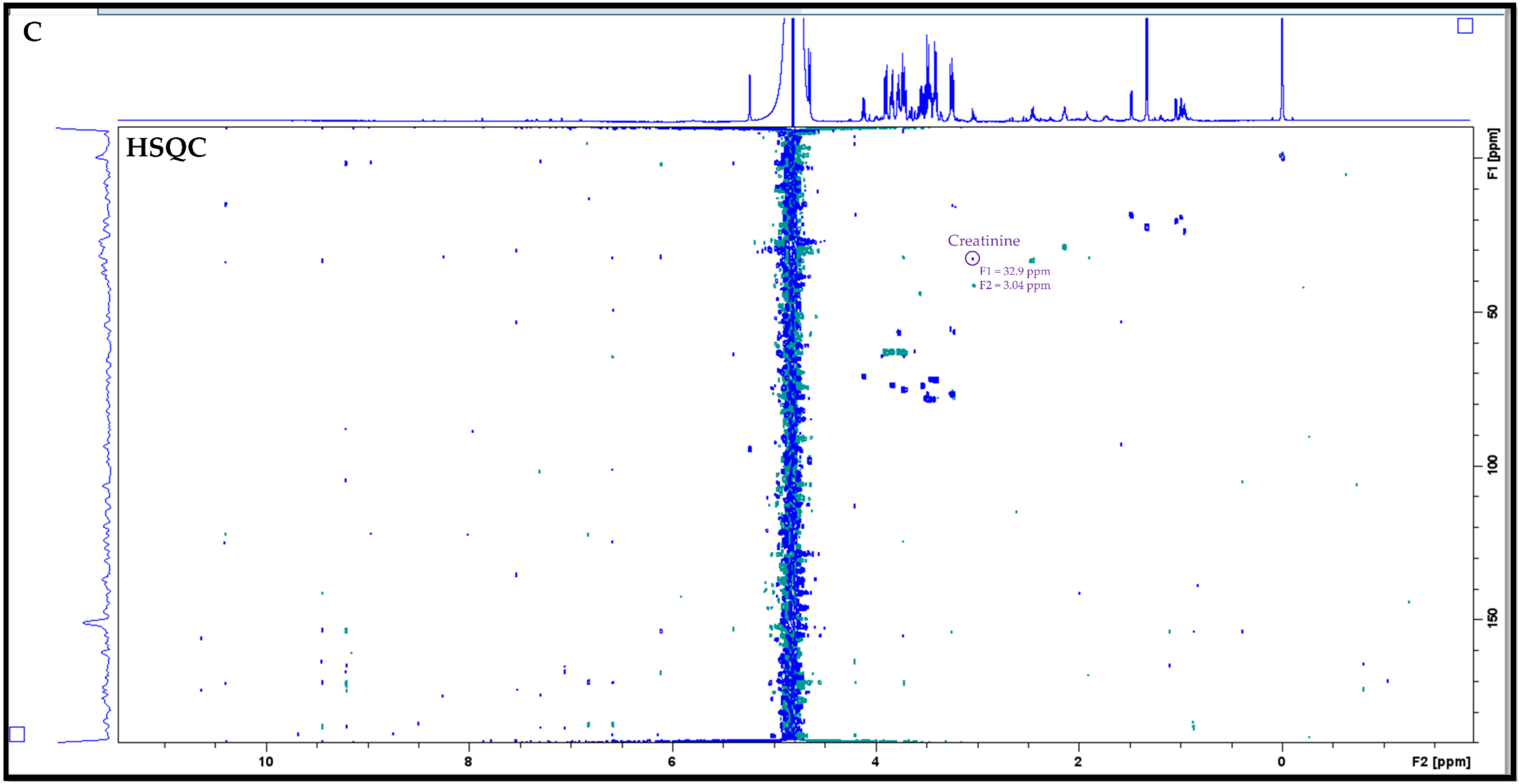

Figure S3. 2D Hetero Single Quantum Coherence (HSQC) experiment for skeletal muscle (**A**) and serum (**C**) samples and TOtal Correlation SpectroscopY (TOCSY) experiment for skeletal muscle sample (**B**) with the most relevant metabolites annotated. No correlation was noted for the serum creatinine and O-acetylcarnitine in the TOCSY experiment.
